# SEX DIFFERENCES BETWEEN ATTITUDES TOWARD MEDICATIONS AND POOR ANTIHYPERTENSIVE MEDICATION ADHERENCE IN ELDERLY

**DOI:** 10.1093/geroni/igz038.971

**Published:** 2019-11-08

**Authors:** Taylor Johnson, Erin Peacock, Julia Silver, James Marsh, Richard Petty, M A Krousel-Wood

**Affiliations:** 1 Tulane University School of Medicine, New Orleans, Louisiana, United States; 2 Tulane University, New Orleans, Louisiana, United States; 3 The Ohio State University, Columbus, Ohio, United States

## Abstract

Despite its importance for blood pressure control, antihypertensive medication adherence remains a challenge in older adults. Explicit and implicit attitudinal ambivalence toward medications (holding both positive and negative explicit attitudes, and discrepant explicit and implicit attitudes, respectively) may underlie low adherence. We examined whether race, age, or sex affect the associations between attitudes, ambivalence, and adherence. A questionnaire and implicit association test captured medication attitudes from hypertensive adults aged ≥55 (n=199). Adherence was measured with the Krousel-Wood Medication Adherence Scale (K-Wood-MAS-4). Higher scores on the attitudes and adherence scales indicate more positive attitudes and worse adherence, respectively. Associations and effect modification by sex, race (white vs. nonwhite), and age (<65 vs. ≥65) were tested in separate ordinary least squares regressions. The sample was 51.0% female, 43.7% nonwhite, 35.5% aged ≥65, with mean K-Wood-MAS-4 0.64 (SD=0.88). Better adherence was associated with more positive net explicit attitudes (β=-0.18, 95% CI -0.30, -0.06, p=0.003), and worse adherence with higher explicit ambivalence (β=-0.05, 95% CI 0.01, 0.09, p=0.028). The associations with explicit attitudes and explicit ambivalence were significant for men (β=-0.30, 95% CI -0.48, -0.11, p=0.002 and β=0.09, 95% CI 0.03, 0.15, p=0.005, respectively) but not for women (β=-0.07, 95% CI -0.423, 0.09, p=0.378 and β=-0.00, 95% CI -0.06, 0.05, p=0.982, respectively) (p-values for interaction=0.062 and 0.031, respectively). No race or age differences were identified. Adherence was not associated with implicit attitudes or implicit ambivalence. In conclusion, explicit attitudes and explicit attitudinal ambivalence may underlie low adherence to antihypertensive medications, particularly for older men.

